# Supplementary material for: Effect of sub-bandgap defects on radiative and non-radiative open-circuit voltage losses in perovskite solar cells
Source: Nat Commun. 2024 Feb 10;15:1276. doi: 10.1038/s41467-024-45512-8 (PMC10858920; doi:10.1038/s41467-024-45512-8)
Supplement: Supplementary file 1 — Supplementary Information [file 41467_2024_45512_MOESM1_ESM.pdf]

Supplementary Information

Effect of sub-bandgap defects on radiative and non-radiative open-circuit voltage losses in perovskite solar cells

Guus J. W. Aalbers[1§], Tom P. A. van der Pol[1§], Kunal Datta[1],
Willemijn H. M. Remmerswaal[1], Martijn M. Wienk[1] & René A. J. Janssen[1,2]*

[1] Molecular Materials and Nanosystems & Institute for Complex Molecular Systems, Eindhoven University of Technology, P.O. Box 513, 5600 MB Eindhoven, The Netherlands.
[2] Dutch Institute for Fundamental Energy Research, De Zaale 20, 5612 AJ Eindhoven, The Netherlands
[§] These authors contributed equally to this work: Guus J. W. Aalbers and Tom P. A. van der Pol.
* e-mail: r.a.j.janssen@tue.nl

Contents Page

Supplementary Figures 1-16 S2
Supplementary Table 1 S16
Supplementary Notes 1-4 S17
Supplementary References S25

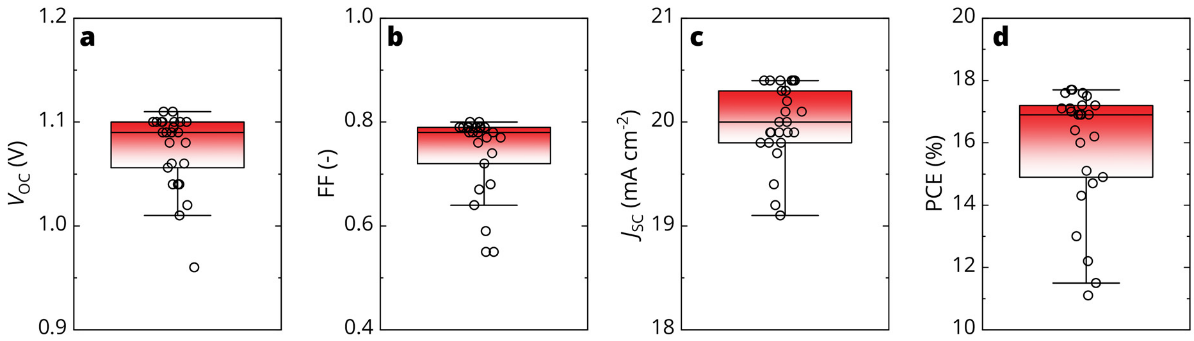

Supplementary Fig. 1 | Statistical distribution of 25 CsFAMA-17 p-i-n devices measured under reverse *J-V* sweeps depicting the **a** V_{OC}, **b** FF, **c** J_{SC}, and **d** PCE. In the boxplots, the median (center black line), 25th and 75th percentile (box limits), and 5th and 95th percentiles (whiskers) are shown.

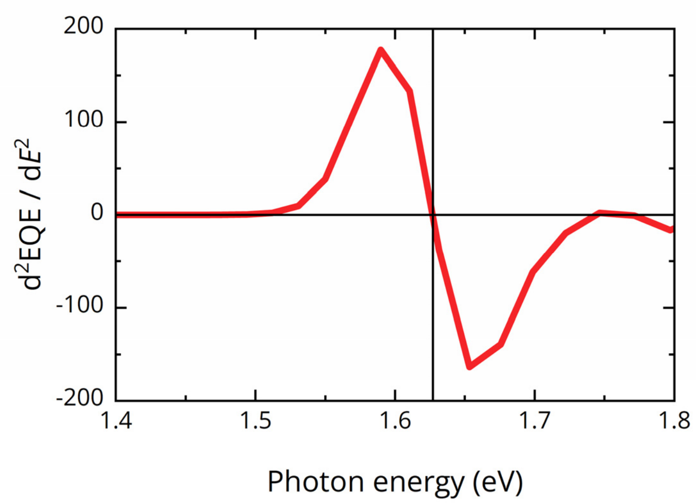

Supplementary Fig. 2 | Optical bandgap determination of CsFAMA-17 p-i-n PSC using the second derivative of the EQE spectrum recorded with 1-sun equivalent bias illumination. When $\frac{\mathrm{d}^2 Q_e^{\mathrm{PV}}}{\mathrm{d}E^2} = 0$, the bandgap is found at 1.627 eV.[1]

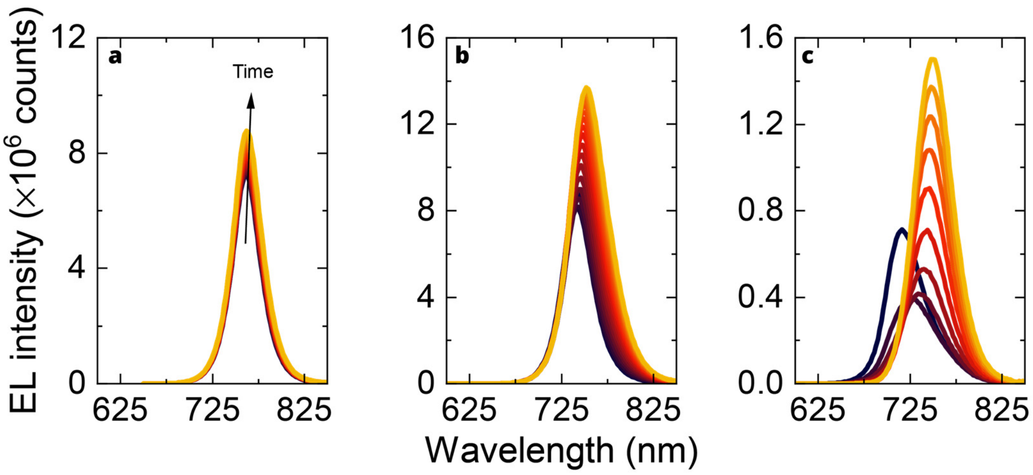

Supplementary Fig. 3 | Time-dependent EL spectra of (K)CsFAMA p-i-n PSCs. a CsFAMA-17 device measured at 2.0 V over 16 min. **b** KCsFAMA-25 device measured at 2.0 V over 20 min. **c** KCsFAMA-40 device measured at 2.5 V over 10 min. Note that the applied biases here are much higher than the 1.5 to 1.7 V applied during regular EL measurements.

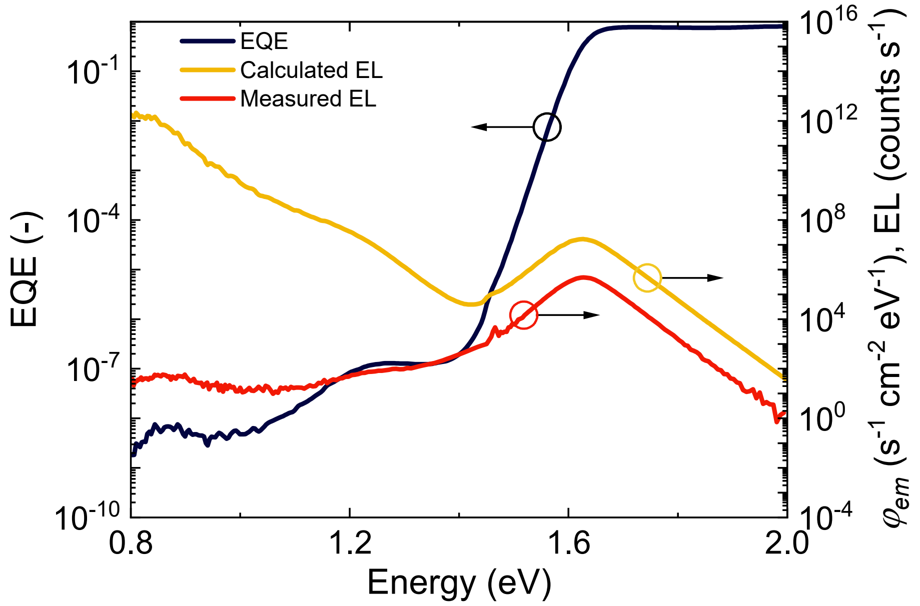

Supplementary Fig. 4 | Measured sensitive EQE spectrum (dark blue) and EL spectrum (red) for a CsFAMA-17 p-i-n PSC. Included is the calculated EL spectrum (φ_{em} in s^{-1} cm^{-2} eV^{-1}, yellow) using the measured EQE spectrum, the reciprocity theorem (equation (6)), and a constant radiative ideality factor ($n_{id}^{rad} = 1$). The calculated EL spectrum diverges from the measured EL spectrum in the region where sub-bandgap defects dominate the EQE spectrum. The small sharp feature in the EL spectrum at ~1.46 eV is a measurement artifact caused by the limited sensitivity of the spectrometer in this spectral range.

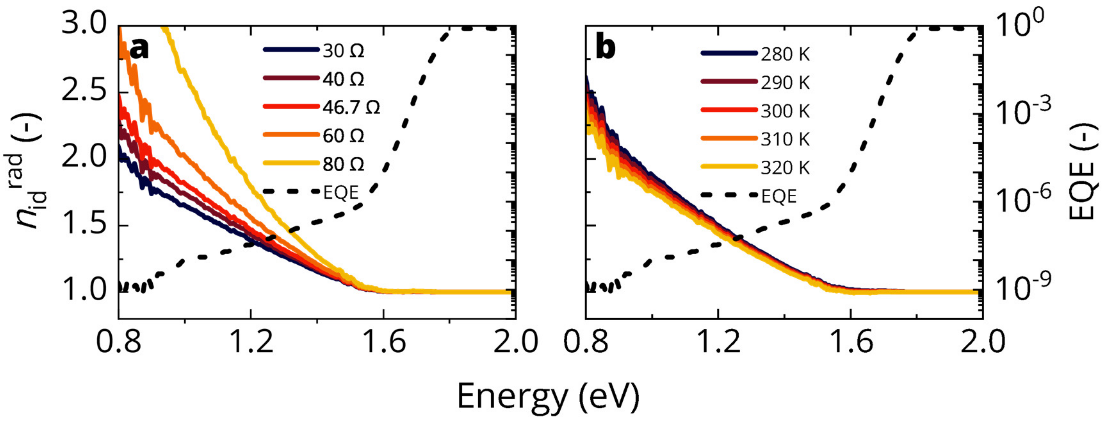

Supplementary Fig. 5 | The calculated energy-dependent radiative ideality factor ($n_{id}^{rad}(E)$) shown for different assumed temperatures and series resistances. After fixing the $n_{id,band}^{rad}$, and measuring the EQE and EL, only the temperature T and the internal voltage V_{int} are unknown in equation (7). $V_{\text{int}} = V - IR_s$, in which V is the applied voltage, I is the measured current, and R_s is the series resistance. **a** Calculated $n_{id}^{rad}(E)$ for a series resistance ranging from 30 to 80 Ω, including the mean R_s (Supplementary Note 1). The corresponding sensitive EQE spectrum is included for reference. The $n_{id}^{rad}(E)$ increases with higher R_s but does not deviate strongly until high resistances (60-80 Ω). The largest differences occur at high energies, where $n_{id}^{rad}(E)$ calculations are in any case more ambiguous as the measured spectra become noise-limited. **b** Calculated $n_{id}^{rad}(E)$ when the temperature is varied from 280 to 320 K and includes the corresponding measured sensitive EQE spectrum. In this temperature range, the $n_{id}^{rad}(E)$ only changes marginally and predominantly at low photon energy.

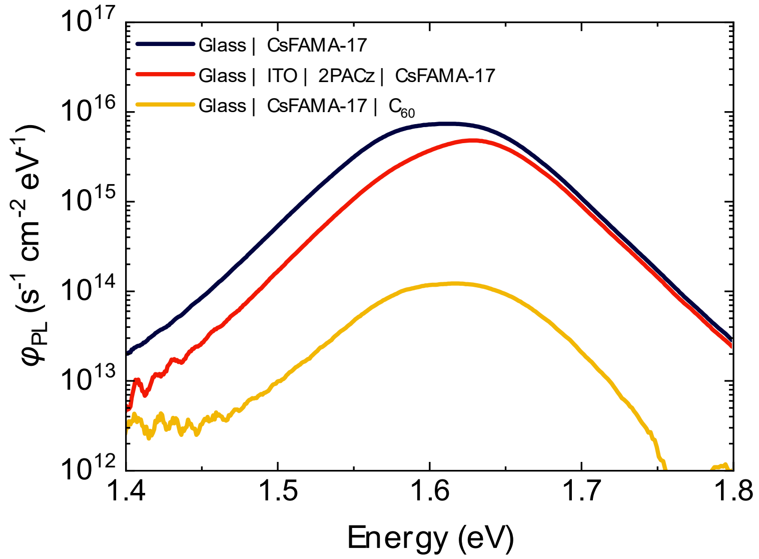

Supplementary Fig. 6 | The absolute photoluminescence spectra for CsFAMA-17 half-stack devices of glass|CsFAMA-17 (dark blue), glass|ITO|2PACz|CsFAMA-17 (red), and glass|CsFAMA-17|C_{60} (orange). The photoluminescence is decreased substantially upon the addition of a C_{60} layer.

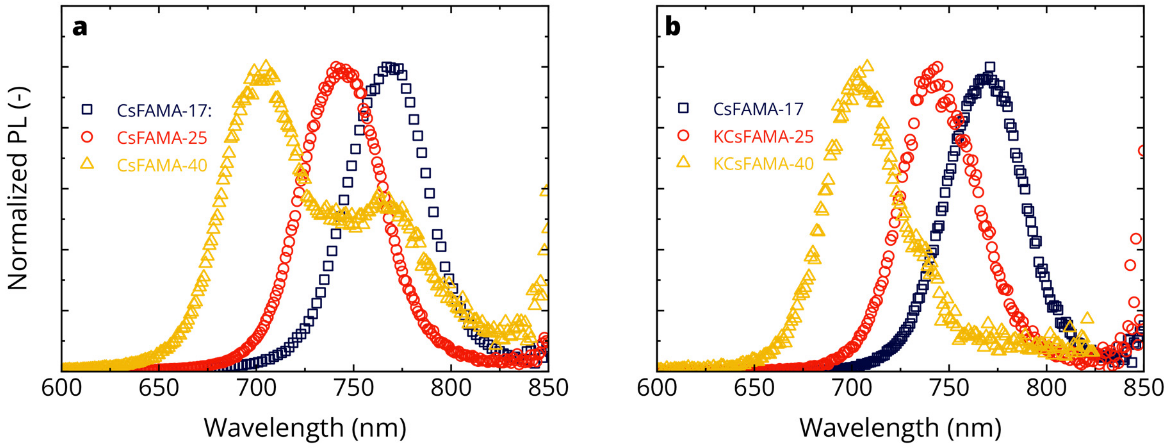

Supplementary Fig. 7 | Normalized PL spectra of (K)CsFAMA-x films show effect of potassium ions on halide seggregation. a CsFAMA-*x* films without 5 vol.% KI, where x = 17, 25, or 40 denotes the bromide fraction, and **b** KCsFAMA-25 and KCsFAMA-40 with 5 vol.% KI additive. When potassium iodide salts are added, the perovskite film is less prone to halide segregation. The experiments are shown for pristine layers, i.e., without prior exposure to light soaking.

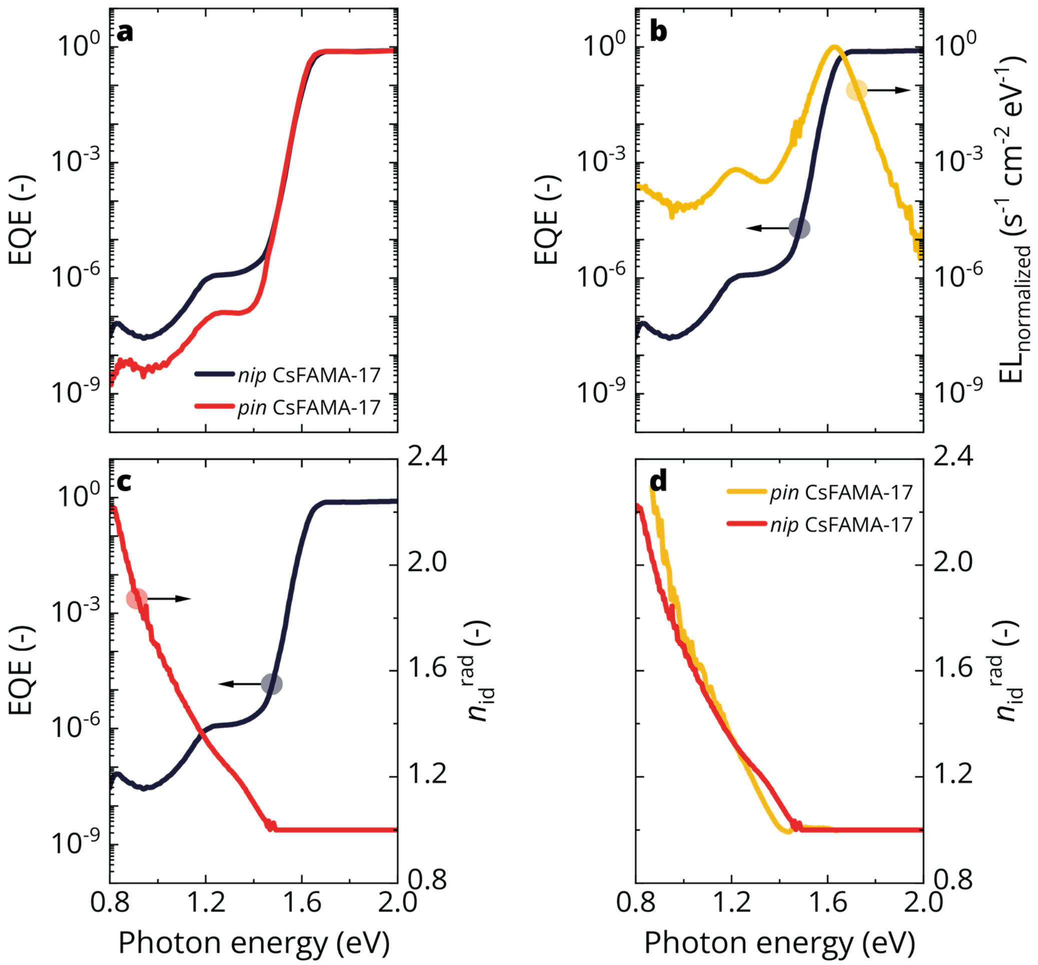

Supplementary Fig. 8 | Defects in CsFAMA-17 PSCs in regular architecture (n-i-p) using the device stack of glass|ITO|SnO_2|PCBM|CsFAMA-17|PM6|MoO_x|Au. a Sensitive EQE spectra on a semilogarithmic scale of an n-i-p device and for comparison also a p-i-n device. **b** EL spectra and **c** $n_{id}^{rad}(E)$ of the n-i-p CsFAMA-17 device. $n_{id}^{rad}(E)$ is 1 above the bandgap until the bottom of the Urbach tail and increases to ~2 for mid-bandgap photon energies. Device parameters: V_{OC} = 1.07 V, FF = 0.75, J_{SC} = 19.6 mA cm^{-2}, PCE = 15.6%, and R_s = 63.2 Ω. **d** Comparison of $n_{id}^{rad}(E)$ for CsFAMA-17 PSCs in p-i-n and n-i-p solar cells.

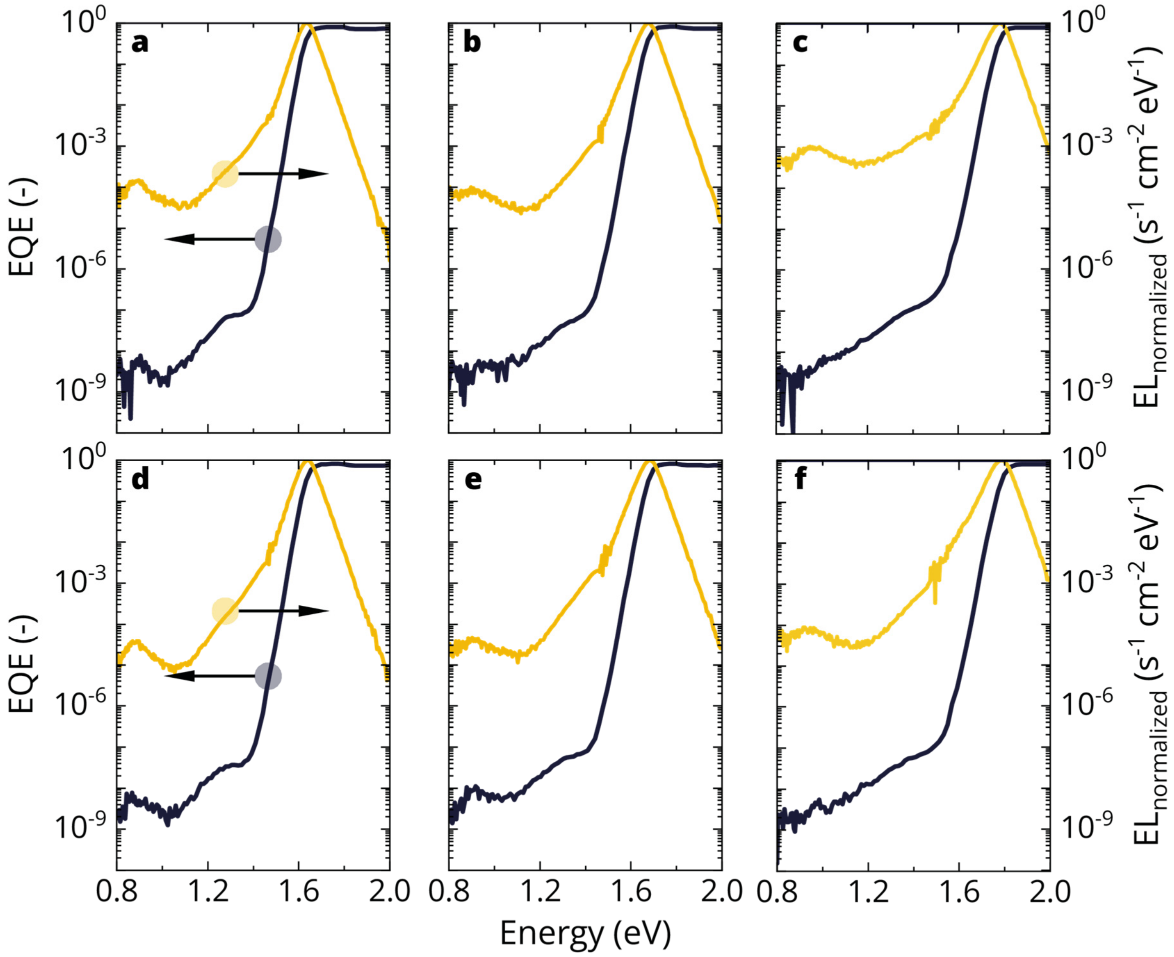

Supplementary Fig. 9 | Sensitive EQE and EL spectra on a semilogarithmic plot for p-i-n CsFAMA-x PSCs. a CsFAMA-17, **b** KCsFAMA-25, **c** KCsFAMA-40, **d** CsFAMA-17 CCl, **e** KCsFAMA-25 CCl, and **f** KCsFAMA-40 CCl. The sharp features in the EL spectra between 1.4 and 1.5 eV are a measurement artifact caused by the limited sensitivity of the spectrometer in this spectral range.

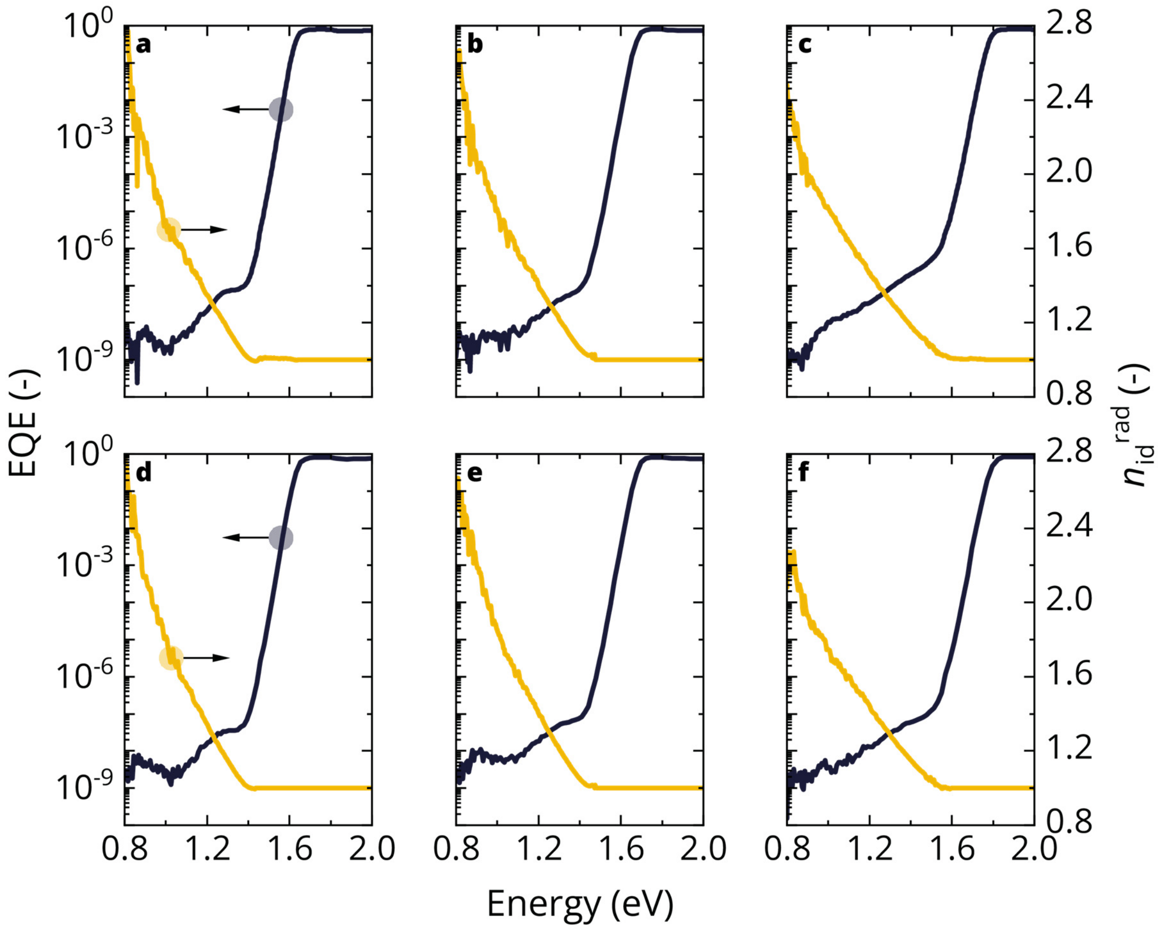

Supplementary Fig. 10 | Sensitive EQE (on a semilogarithmic plot) and $n_{id}^{rad}(E)$ spectra for p-i-n CsFAMA-x PSCs. **a** CsFAMA-17, **b** KCsFAMA-25, **c** KCsFAMA-40, **d** CsFAMA-17 CCl, **e** KCsFAMA-25 CCl, and **f** KCsFAMA-40 CCl. All compositions show a $n_{id}^{rad}(E)$ that is 1 along the Urbach tail and increases to ~2 for mid-bandgap photon energies.

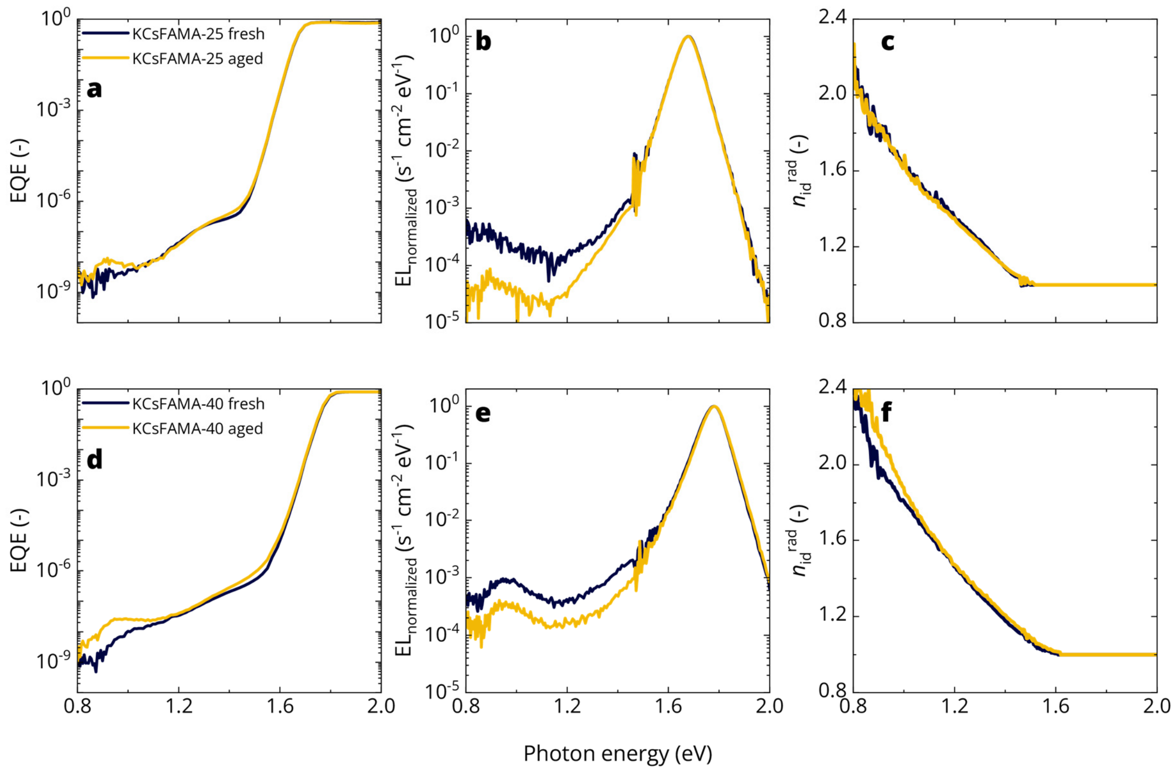

Supplementary Fig. 11 | Sub-bandgap response of three-months aged KCsFAMA-25 and KCsFAMA-40 PSCs. **a** Sensitive EQE and **b** EL spectra on a semilogarithmic scale and **c** $n_{\mathrm{id}}^{\mathrm{rad}}(E)$ of fresh and aged KCsFAMA-25 device. **d** Sensitive EQE and **e** EL spectra on a semilogarithmic scale and **f** $n_{\mathrm{id}}^{\mathrm{rad}}(E)$ of fresh and aged KCsFAMA-40 device. $n_{\mathrm{id}}^{\mathrm{rad}}(E)$ is 1 above the bandgap until the bottom of the Urbach tail and increases to ~2 for mid-bandgap photon energies.

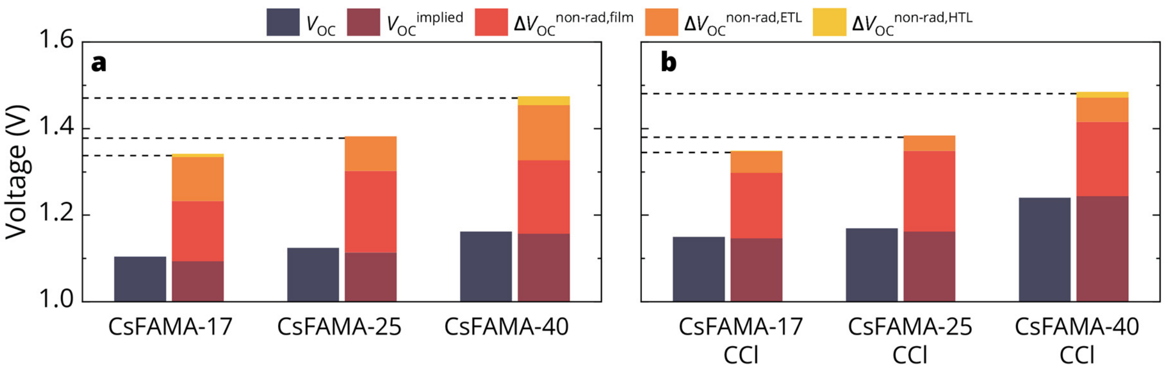

Supplementary Fig. 12 | Disentanglement of voltage losses for CsFAMA-*x* p-i-n PSCs using absolute photoluminescence and the many-diode model. a For CsFAMA-17, KCsFAMA-25, and KCsFAMA-40 p-i-n PSCs. **b** For CsFAMA-17 CCl, KCsFAMA-25 CCl, and KCsFAMA-40 CCl p-i-n PSCs. The V_{OC}^{rad} is determined using the many-diode model (dashed line), and V_{OC} in the left bar is measured from *J*-*V* sweeps. $\Delta V_{OC}^{non\text{-}rad,film}$, $\Delta V_{OC}^{non\text{-}rad,ETL}$, and $\Delta V_{OC}^{non\text{-}rad,HTL}$ are determined from QFLS measurements according to equation (11) and (12), respectively. The $V_{OC}^{implied}$ in the right bar is calculated as V_{OC}^{rad} minus $\Delta V_{OC}^{non\text{-}rad,film}$, $\Delta V_{OC}^{non\text{-}rad,ETL}$, and $\Delta V_{OC}^{non\text{-}rad,HTL}$ and can slightly deviate from the measured average V_{OC} which we attribute to inaccuracies in the determination of QFLS.

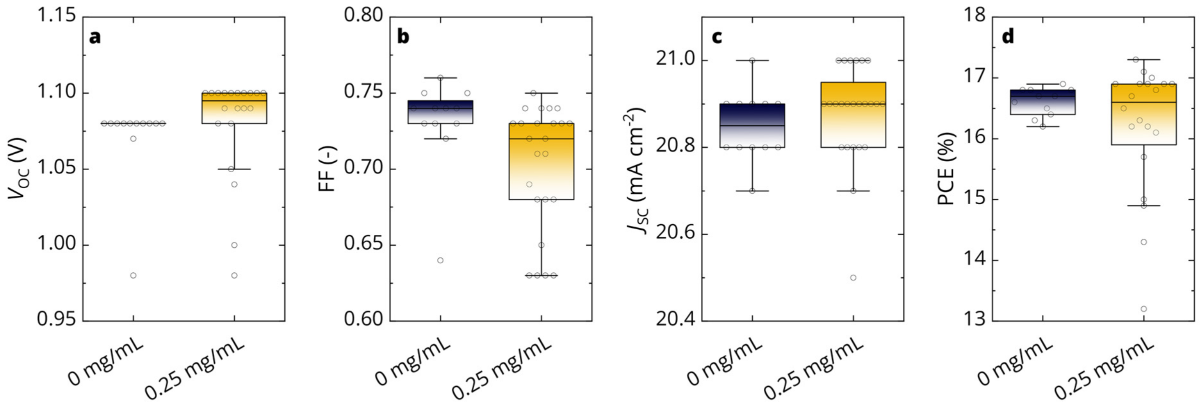

Supplementary Fig. 13 | Statistical distribution of 20 CsFAMA-17 p-i-n devices with or without phenformin hydrochloride grain boundary passivation measured under reverse *J-V* sweeps depicting the **a** V_{OC}, **b** fill factor, **c** J_{SC}, and **d** PCE. In the boxplots, the median (center black line), 25th and 75th percentile (box limits), and 5th and 95th percentiles (whiskers) are shown.

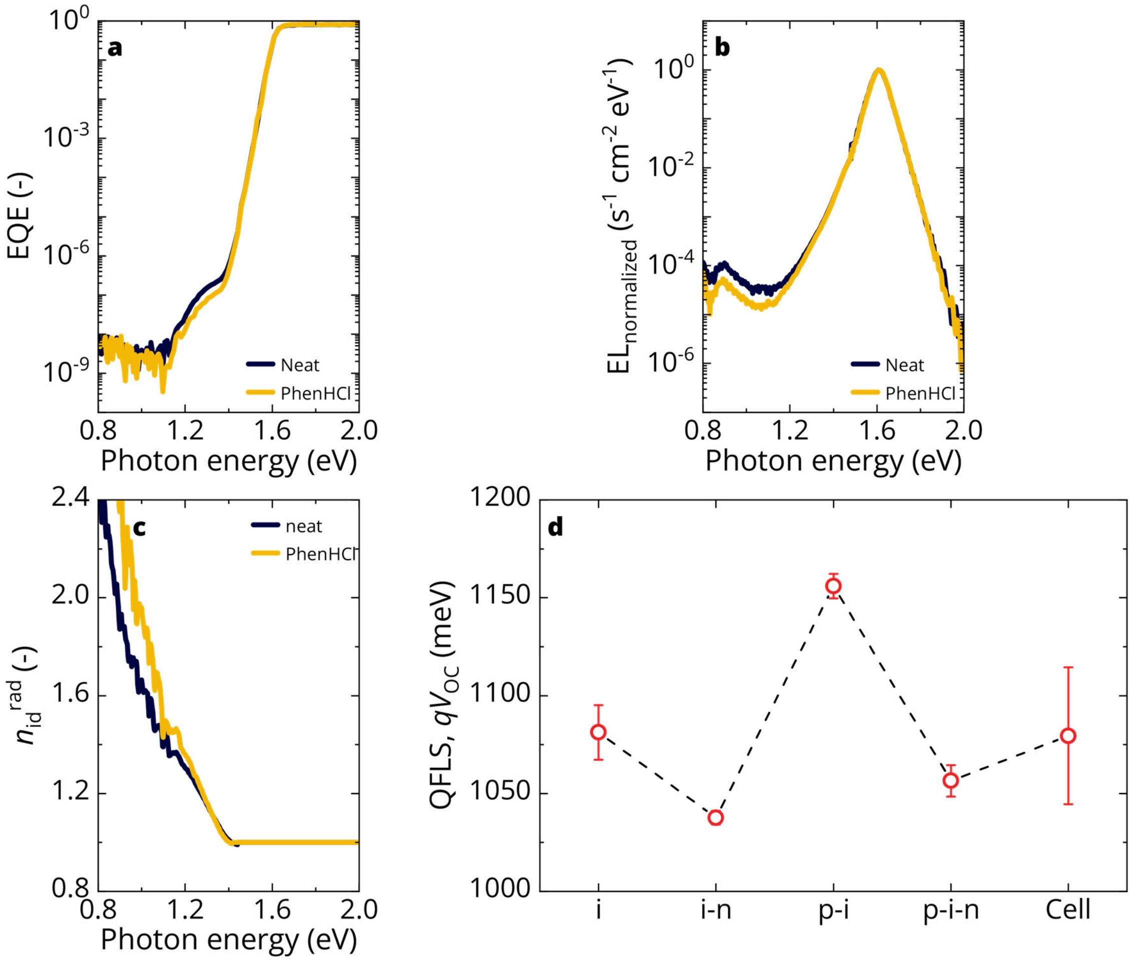

Supplementary Fig. 14 | Voltage loss analysis of phenformin hydrochloride (PhenHCl) bulk additive passivated CsFAMA-17 PSCs. Sensitive **a** EQE and **b** EL spectra of neat CsFAMA-17 and PhenHCl bulk passivated PSC (CsFAMA-17 PhenHCl) with 0.25 mg/mL PhenHCl in the perovskite precursor solution. **c** Comparison of $n_{\mathrm{id}}^{\mathrm{rad}}(E)$ for neat CsFAMA-17 and CsFAMA-17 PhenHCl devices. $n_{\mathrm{id}}^{\mathrm{rad}}(E)$ is 1 above the bandgap until the bottom of the Urbach tail and increases to ~2 for mid-bandgap photon energies. **d** Quasi-Fermi level splitting values of CsFAMA-17 PhenHCl partial stacks of glass|perovskite (i), glass|perovskite|C_{60} (i-n), glass|ITO|2PACz|perovskite (p-i), and glass|ITO|2PACz|perovskite|C_{60} (p-i-n) under 1 Sun illumination. Experimental external V_{OC} of CsFAMA-17 PhenHCl device added for reference (cell).

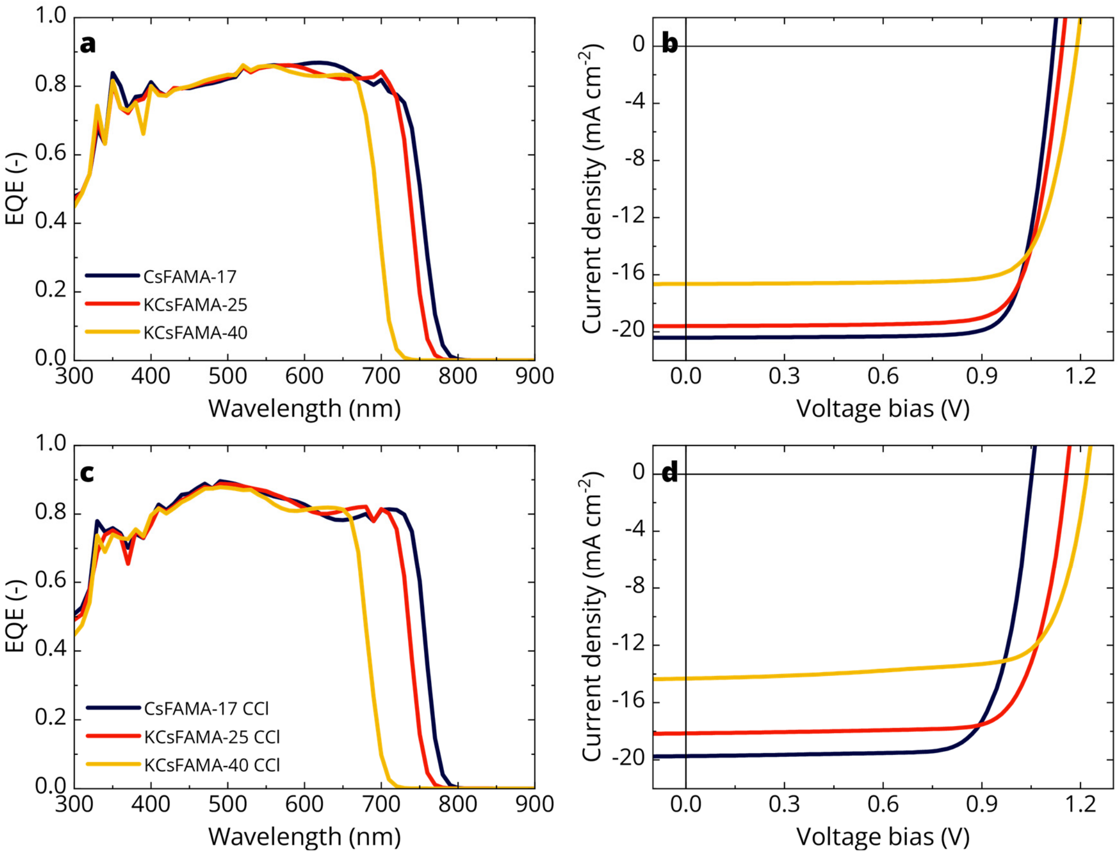

Supplementary Fig. 15 | Spectral response and *J-V* behavior of neat and choline chloride (CCl) passivated p-i-n CsFAMA-x PSCs. EQE spectra recorded for **a** ITO|2PACz|CsFAMA-*x*|C_{60}|BCP|Al and **c** ITO|2PACz|CsFAMA-*x*|CCl|C_{60}|BCP|Al with 1-sun equivalent bias illumination, where *x* is the bromide fraction. *J-V* measurements in the reverse scan direction of **b** ITO|2PACz|CsFAMA-*x*|C_{60}|BCP|Al and **d** ITO|2PACz|CsFAMA-*x*|CCl|C_{60}|BCP|Al devices measured with simulated AM1.5G (100 mW cm^{-2}) illumination.

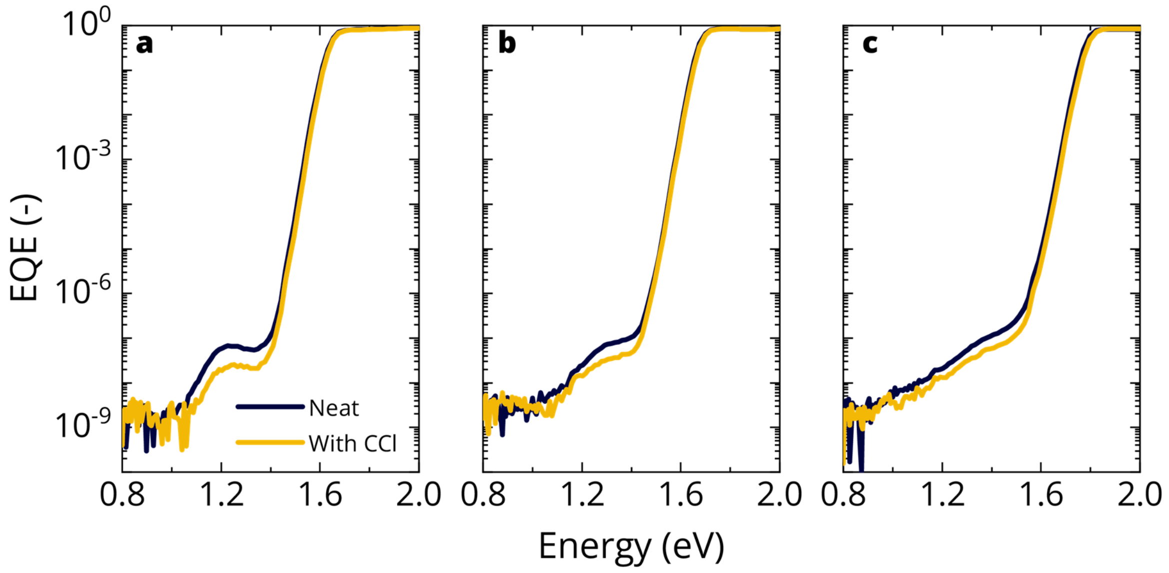

Supplementary Fig. 16 | A comparison of sensitive EQE spectra recorded with/without CCl passivation (yellow/dark blue, respectively). a CsFAMA-17, **b** KCsFAMA-25, and **c** KCsFAMA-40 *p-i-n* PSCs. For all compositions, the defect response decreases after CCl passivation.

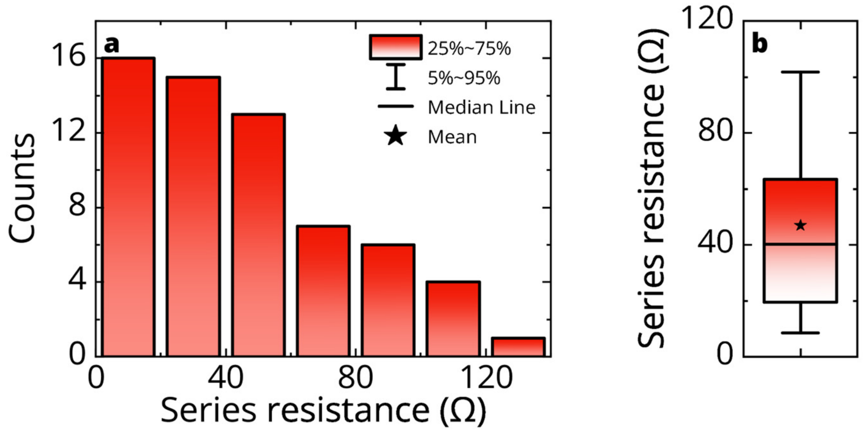

Supplementary Fig. 17 | Statistical analysis of series resistance (R_s) of 64 *p-i-n* CsFAMA PSCs. a Distribution R_s CsFAMA devices calculated using equation S1. **b** The mean series resistance is 46.7 Ω and the median is 40 Ω.

Supplementary Table 1 | Voltage loss analysis of three-months aged KCsFAMA-25 and KCsFAMA-40 PSCs showing bandgap (E_g), V_{OC}^{rad}, V_{OC}, $\Delta V_{OC}^{non-rad}$, and Urbach energy (E_U) before and after 3 months of aging. V_{OC}^{rad} and $\Delta V_{OC}^{non-rad}$, determined using the many-diode model and V_{OC} experimentally measured.

	E_g (eV)	V_{OC}^{rad} (V)	V_{OC} (V)	$\Delta V_{OC}^{non-rad}$ (mV)	E_U (meV)
KCsFAMA-25	1.69	1.385	1.11	275	14.5
KCsFAMA-40	1.79	1.479	1.17	309	16.2
KCsFAMA-25 aged	1.69	1.383	1.11	273	14.4
KCsFAMA-40 aged	1.78	1.478	1.08	398	16.2

Supplementary Note 1 | Internal voltage (V_{int})

To calculate the energy-dependent radiative ideality factor, it is crucial to accurately determine the internal voltage (V_{int}) of the PSC. The V_{int} is the bias applied over the active layer and is defined as $V_{\text{int}} = V - IR_{\text{s}}$, in which V is the applied voltage, I is the measured current, and R_{s} is the series resistance. The accuracy of V_{int} is limited by the determination of series resistance (R_{s}) since V and I can easily be measured. Pysch et al.[2] have developed a method to calculate R_{s} using the *J-V* characteristics of a solar cell in the dark and under illumination. Note that the current density (*J*) is obtained from the current (*I*) by dividing it by the cell area. In their approach, Pysch et al. translate a dark *J-V* curve by the short-circuit current density (J_{SC}) under AM1.5G following $J_{\text{dark}}(V) - J_{\text{SC}}$. This gives rise to a maximum power point and a V_{OC} in the dark ($V_{\text{OC}}^{\text{dark}}$). Then they propose a formula for the R_{s} given as

$$R_{\text{s}} = \frac{V_{\text{MPP}}^{\text{dark}} - V_{\text{MPP}}^{\text{light}} - (|J_{\text{SC}}| - |J_{\text{MPP}}|)R_{\text{s}}^{\text{dark}}}{|J_{\text{MPP}}|} \tag{S1}$$

with,

$$R_{\text{s}}^{\text{dark}} = \frac{V_{\text{OC}}^{\text{dark}} - V_{\text{OC}}}{|J_{\text{SC}}|} \tag{S2}$$

Here, $V_{\text{MPP}}^{\text{dark}}$ is the voltage in the maximum power point of the shifted dark *J-V* curve, $V_{\text{MPP}}^{\text{light}}$ the maximum power point voltage under AM1.5G illumination, J_{MPP} the current density in the maximum power point under AM1.5G, $R_{\text{s}}^{\text{dark}}$ the series resistance in the dark, and $V_{\text{OC}}^{\text{dark}}$ the open-circuit voltage of the shifted dark *J-V* curve. From this equation, we calculate a mean series resistance of 46.7 Ω for 64 CsFAMA devices (Supplementary Fig. 14).

Supplementary Note 2 | Derivation of energy-dependent ideality factor

For the derivation of the formula used to calculate the energy-dependent ideality factor (equation (7) in the main text), we start with Rau's optoelectronic reciprocity theorem that relates EQE to EL.[3] From there we obtain

$$\varphi_{\mathrm{em}}(E) = \varphi_{\mathrm{bb}}(E)\, Q_e^{\mathrm{PV}}(E) \left\{ exp\left(\frac{qV_{\mathrm{int}}}{n_{id}^{rad} k_{\mathrm{B}} T} \right) - 1 \right\} \qquad (S3)$$

Here, $\varphi_{\mathrm{em}}(E)$ is the EL emission photon flux, $\varphi_{\mathrm{bb}}(E)$ is the black-body radiation photon flux at room temperature, and $Q_e^{\mathrm{PV}}(E)$ is the EQE spectrum as a function of photon energy (E). V_{int} is the internal voltage (see also Supplementary Note 1), q is the elementary charge, and $n_{\mathrm{id}}^{\mathrm{rad}}$ is the radiative ideality factor. We note that the reciprocity relation does not hold over the entire spectrum, as described in the main text and visualized in Supplementary Fig. 2. Such strong deviations of several orders of magnitude are likely the result of a changing ideality factor. We, therefore, propose an energy-dependent ideality factor $n_{\mathrm{id}}^{\mathrm{rad}}(E)$ inspired by the double-diode model as outlined in the main text. Then we describe the reciprocity of EL emission at two different photon energies, namely above-bandgap (band) and sub-bandgap (defect) as follows

$$\varphi_{\mathrm{em}}^{\mathrm{band}}(E_{band}) = \varphi_{\mathrm{bb}}^{\mathrm{band}}(E_{\mathrm{band}}) Q_{e,\mathrm{band}}^{\mathrm{PV}}(E_{\mathrm{band}}) \left\{ \exp\left(\frac{qV_{\mathrm{int}}}{n_{\mathrm{id,band}}^{\mathrm{rad}}(E_{\mathrm{band}}) k_{\mathrm{B}} T} \right) - 1 \right\} \qquad (S4)$$

$$\varphi_{\mathrm{em}}^{\mathrm{defect}}(E_{\mathrm{defect}}) = \varphi_{\mathrm{bb}}^{\mathrm{defect}}(E_{\mathrm{defect}}) Q_{e,\mathrm{defect}}^{\mathrm{PV}}(E_{\mathrm{defect}}) \left\{ \exp\left(\frac{qV_{\mathrm{int}}}{n_{\mathrm{id,defect}}^{\mathrm{rad}}(E_{\mathrm{defect}}) k_{\mathrm{B}} T} \right) - 1 \right\} \qquad (S5)$$

We fix the E_{band} as the energy of the emission maximum (E_{max}), denoting all parameters as constants (e.g., $\varphi_{\mathrm{em}}^{\mathrm{band}}(E_{\mathrm{max}}) = \varphi_{\mathrm{em}}^{\mathrm{band}}$). By dividing the reciprocity relation for above-bandgap and sub-bandgap transitions, we obtain

$$\frac{\varphi_{\mathrm{em}}^{\mathrm{band}}}{\varphi_{\mathrm{em}}^{\mathrm{defect}}(E_{\mathrm{defect}})} = \frac{\varphi_{\mathrm{bb}}^{\mathrm{band}} Q_{e,\mathrm{band}}^{\mathrm{PV}} \left\{ \exp\left(\frac{qV_{\mathrm{int}}}{n_{\mathrm{id,band}}^{\mathrm{rad}} k_{\mathrm{B}} T} \right) - 1 \right\}}{\varphi_{\mathrm{bb}}^{\mathrm{defect}}(E_{\mathrm{defect}}) Q_{e,\mathrm{defect}(E_{\mathrm{defect}})}^{\mathrm{PV}} \left\{ \exp\left(\frac{qV_{\mathrm{int}}}{n_{\mathrm{id,defect}}^{\mathrm{rad}}(E_{\mathrm{defect}}) k_{\mathrm{B}} T} \right) - 1 \right\}} \qquad (S6)$$

The sub/super-script "defect" was then omitted, as this relation should hold for any energy

$$\frac{\varphi_{\mathrm{em}}^{\mathrm{band}}}{\varphi_{\mathrm{em}}(E)} = \frac{\varphi_{\mathrm{bb}}^{\mathrm{band}} Q_{e,\mathrm{band}}^{\mathrm{PV}} \left\{ \exp\left(\frac{qV_{\mathrm{int}}}{n_{\mathrm{id,band}}^{\mathrm{rad}} k_{\mathrm{B}} T} \right) - 1 \right\}}{\varphi_{\mathrm{bb}}(E) Q_e^{\mathrm{PV}}(E) \left\{ \exp\left(\frac{qV_{\mathrm{int}}}{n_{\mathrm{id}}^{\mathrm{rad}}(E) k_{\mathrm{B}} T} \right) - 1 \right\}} \qquad (S7)$$

In this equation, all parameters can be experimentally accessed except for the ideality factors. Thereafter, we rearrange to free $n_{\mathrm{id}}^{\mathrm{rad}}(E)$ while neglecting the -1 terms yielding equation (7) in the main text

$$n_{\mathrm{id}}^{\mathrm{rad}}(E) = \left[\frac{1}{n_{\mathrm{id,band}}^{\mathrm{rad}}} - \frac{k_{\mathrm{B}}T}{qV_{\mathrm{int}}} \ln\left(\frac{\varphi_{\mathrm{em}}^{\mathrm{band}} \cdot \varphi_{\mathrm{bb}}^{\mathrm{defect}}(E) \cdot Q_{e,\mathrm{defect}}^{\mathrm{PV}}(E)}{\varphi_{\mathrm{em}}^{\mathrm{defect}}(E) \cdot \varphi_{\mathrm{bb}}^{\mathrm{band}} \cdot Q_{e,\mathrm{band}}^{\mathrm{PV}}}\right)\right]^{-1} \quad \text{(S8)}$$

This equation allows us to calculate the ideality factor as a function of photon energy from the EQE and EL spectrum if the ideality factor at an arbitrary position (in this case corresponding to the EL maximum) is known.

Supplementary Note 3 | Varying ideality factor

In the SRH model, the defect state lies exactly at mid-bandgap energy resulting in a similar capture coefficient for electrons and holes. As the electron and hole concentration are also equal, recombination for electrons and holes has identical rates and the defect can be regarded as always-filled. Thus in SRH recombination, the recombination rate is independent of the filling rate of the defect. If, however, the defect state energy deviates from mid-bandgap energy, the transition to the valence band and conduction band differ in energy. The highest energy transition can be expected to be rate limiting and the filling rate of one carrier type in the defect influences the overall rate. The recombination rate in the defect then also becomes dependent on the charge carrier density of this carrier type and therefore is no longer 1st order. This can be readily found from the SRH expression of the recombination rate on a defect, expressed as

$$R = \frac{c_{\mathrm{F}n} c_{\mathrm{F}p} N_{\mathrm{T}} (np - p_i n_i)}{c_{\mathrm{F}p}\left(p + p_i \frac{f}{1-f}\right) + c_{\mathrm{F}n}\left(n + n_i \frac{1-f}{f}\right)} \tag{S9}$$

Where c_{F} is the electron/hole capture rate, N_{T} is the defect density, n and p are the electron and hole density respectively, while n_i and p_i are the electron/hole density at thermal equilibrium and f is the fraction of defects filled with electrons under thermal equilibrium. Generally, $p_i \ll p$ and $n_i \ll n$ and for mid-bandgap states the $f \sim 0.5$ and thus also $p_i \frac{f}{1-f} \ll p$ and $n_i \frac{1-f}{f} \ll n$. If $c_{\mathrm{F}n} = c_{\mathrm{F}p} = c_{\mathrm{F}}$ while $p \sim n$, the recombination rate simplifies to $R = c_{\mathrm{F}} N_{\mathrm{T}} n$ yielding an ideality factor of 2 ($n_{\mathrm{id}} = 2$).

Now, let us assume a defect positioned close to the valence band. For this defect, the transition from the defect to the conduction band will be rate limiting ($c_{\mathrm{F}n} \ll c_{\mathrm{F}p}$) and $np \gg p_i n_i$, yielding

$$R = \frac{c_{\mathrm{F}n} c_{\mathrm{F}p} N_{\mathrm{T}} (np)}{c_{\mathrm{F}p}\left(p + p_i \frac{f}{1-f}\right)} \tag{S10}$$

For this defect, $f \rightarrow 1$ and therefore $p_i \frac{f}{1-f} \gg p$. With $n \sim p$, this simplifies the equation to

$$R = \frac{c_{\mathrm{F}n} N_{\mathrm{T}} p^2}{p_0 \frac{f}{1-f}} \tag{S11}$$

Which is 2nd order in p and thus yields an n_{id} of 1, similar to BTB recombination. An equivalent derivation can of course be performed for a defect located near the conduction band predominantly filled with holes ($f \rightarrow 0$).

An explicit description of the fraction of electron-filled defects (f) follows

$$f = \frac{1}{1 + \exp\left(\frac{E_{\mathrm{T}} - E_{\mathrm{F}}}{k_{\mathrm{B}}T}\right)} \tag{S12}$$

Where E_{T} and E_{F} are the defect and Fermi energy respectively. The well-known expression for the hole density reads

$$p = p_i \exp\left(\frac{E_{\mathrm{F}} - E_{\mathrm{F}p}}{k_B T}\right) \tag{S13}$$

with $E_{\mathrm{F}p}$ the quasi-Fermi level for holes. The denominator in the recombination rate equation for a defect close to the valence band then becomes

$$c_{\mathrm{F}p}\left(p + p_i \frac{f}{1-f}\right) = c_{\mathrm{F}p} p_i \left\{ \exp\left(\frac{E_{\mathrm{F}} - E_{\mathrm{F}p}}{k_B T}\right) + \frac{\frac{1}{1 + \exp\left(\frac{E_{\mathrm{T}} - E_{\mathrm{F}}}{k_{\mathrm{B}}T}\right)}}{1 - \frac{1}{1 + \exp\left(\frac{E_{\mathrm{T}} - E_{\mathrm{F}}}{k_{\mathrm{B}}T}\right)}} \right\} \tag{S14}$$

This expression cannot be simplified to $\left(\exp\left[\frac{E_{\mathrm{F}} - E_{\mathrm{F}p}}{k_{\mathrm{B}}T}\right]\right)^a$ and thus p^a, but there are cases where it can be approximated as such. Necessarily, the a is dependent on E_{T}. We note that for defect states close to mid-bandgap, the approximation $c_{\mathrm{F}n} \ll c_{\mathrm{F}p}$ or $c_{\mathrm{F}n} \gg c_{\mathrm{F}p}$ does not hold, and the original SRH expression cannot be simplified.

In conclusion, with $f(E_{\mathrm{T}})$ determining the relative influence of p and $p_i \frac{f}{1-f}$ (and similarly n and $n_i \frac{1-f}{f}$) in the denominator, the ideality factor will vary according to the defect energy as was observed in our work.

We note that the theoretical derivation outlined above is not limited to perovskite solar cells. We envision that similar E_{T}-dependent radiative ideality factors describe recombination at defect sites in other systems, but have so far not been observed due to the difficulty of measuring EQE and EL stemming from these states.

Supplementary Note 4 | Electroluminescence quantum efficiency and non-radiative voltage loss in the many-diode model

We describe the electroluminescence quantum efficiency (Q_e^{EL}), and its relation to non-radiative voltage loss ($\Delta V_{\mathrm{OC}}^{\text{non-rad}}$), in the many-diode model following the derivation of Rau (*Phys. Rev. B* **76**, 085303 (2007)).

Q_e^{EL} is determined by the ratio of the emissive recombination current density (J_{em}) and the total injected current density (J_{inj})

$$Q_e^{\mathrm{EL}} = \frac{J_{\mathrm{em}}}{J_{\mathrm{inj}}} \tag{S15}$$

In the many-diode model, the emissive recombination is a summation of emission recombination for each individual diode with characteristic energy E ($J_{\mathrm{em}} = \sum J^{\mathrm{rad}}(E_i)$). Thus equation (S15) becomes

$$Q_e^{\mathrm{EL}} = \frac{\sum J^{\mathrm{rad}}(E_i)}{J_{\mathrm{inj}}} \tag{S16}$$

Note that $\sum J^{\mathrm{rad}}(E_i)$ is dependent on V_{int} and not limited to $V_{\mathrm{OC}}^{\mathrm{rad}}$ here. We conclude from our results regarding the radiative ideality factor, shown in Figure 2b, that the many-diodes can be divided into a set of diodes with (1) constant and (2) energy dependent $n_{\mathrm{id}}^{\mathrm{rad}}$, respectively. Similarly, we divide the emissive recombination into two summations for constant $n_{\mathrm{id}}^{\mathrm{rad}}$ ($n_{\mathrm{id},1}^{\mathrm{rad}}$ and $\sum J_1^{\mathrm{rad}}(E_i)$) and for an energy-dependent $n_{\mathrm{id}}^{\mathrm{rad}}$ ($n_{\mathrm{id},2}^{\mathrm{rad}}(E_i)$ and $\sum J_2^{\mathrm{rad}}(E_i)$). Further, the current contribution to the EL spectrum of states with an energy-dependent radiative ideality factor is negligible at $V_{\mathrm{int}} \sim V_{\mathrm{OC}}$ ($\sum J_1^{\mathrm{rad}}(E_i) \gg \sum J_2^{\mathrm{rad}}(E_i)$) as is evident from Figure 2 and 3c, thereby equation (S16) transforms to

$$Q_e^{\mathrm{EL}} = \frac{\sum J_1^{\mathrm{rad}}(E_i) + \sum J_2^{\mathrm{rad}}(E_i)}{J_{\mathrm{inj}}} = \frac{\sum J_1^{\mathrm{rad}}(E_i)}{J_{\mathrm{inj}}} \tag{S17}$$

At V_{int} far below V_{OC}, assuming $\sum J_1^{\mathrm{rad}}(E_i) \gg \sum J_2^{\mathrm{rad}}(E_i)$ does not hold and equation (S17) is no longer valid. However, commonly EL and conversely Q_e^{EL} is not (and cannot) be measured at low V_{int} and corresponding J_{inj}.

We can simplify the expression for $V_{\mathrm{OC}}^{\mathrm{rad}}$ in an analogous manner. Starting from equation (9) in the main text and neglecting the -1 term, we split the summation into a (1) constant and (2) energy-dependent $n_{\mathrm{id}}^{\mathrm{rad}}$ with appropriate current contributions ($J_{\Delta E,1}^{\mathrm{rad}}(E_i)$ and $J_{\Delta E,2}^{\mathrm{rad}}(E_i)$, respectively)

$$-J_{\mathrm{ph}} + \sum J_{\Delta E,1}^{\mathrm{rad}}(E_i)\left\{\exp\left(\frac{qV_{\mathrm{OC}}^{\mathrm{rad}}}{n_{\mathrm{id},1}^{\mathrm{rad}}k_{\mathrm{B}}T}\right)\right\} + \sum J_{\Delta E,2}^{\mathrm{rad}}(E_i)\left\{\exp\left(\frac{qV_{\mathrm{OC}}^{\mathrm{rad}}}{n_{\mathrm{id},2}^{\mathrm{rad}}(E_i)k_{\mathrm{B}}T}\right)\right\} = 0 \quad \text{(S18)}$$

Here $\exp\left(\frac{qV_{\mathrm{OC}}^{\mathrm{rad}}}{n_{\mathrm{id},1}^{\mathrm{rad}}k_{\mathrm{B}}T}\right)$ is independent of E and, from the data in Figure 2b, $n_{\mathrm{id},1}^{\mathrm{rad}}$ is equal to 1 in our case. Since we have shown that the contributed current from $n_{\mathrm{id},2}^{\mathrm{rad}}(E_i)$ states is negligible at $V_{\mathrm{OC}}^{\mathrm{rad}}$ (Figure 3c), we can simplify equation (S18) to

$$-J_{\mathrm{ph}} + J_{0,1}^{\mathrm{rad}} \exp\left(\frac{qV_{\mathrm{OC}}^{\mathrm{rad}}}{n_{\mathrm{id},1}^{\mathrm{rad}}k_{\mathrm{B}}T}\right) = 0 \quad \text{(S19)}$$

with $\sum J_{\Delta E,1}^{\mathrm{rad}}(E_i) = J_{0,1}^{\mathrm{rad}}$. In general, emissive recombination for states with $n_{\mathrm{id},1}^{\mathrm{rad}}$ is given by

$$\sum J_1^{\mathrm{rad}}(E_i) = J_{0,1}^{\mathrm{rad}} \exp\left(\frac{qV_{\mathrm{int}}}{n_{\mathrm{id},1}^{\mathrm{rad}}k_{\mathrm{B}}T}\right) \quad \text{(S20)}$$

If we then evaluate the natural logarithm of equation (S17) at V_{OC}, and insert equation (S20), we obtain

$$\ln(Q_e^{\mathrm{EL}}) = \ln\left(\frac{J_{0,1}^{\mathrm{rad}} \exp\left(\frac{qV_{\mathrm{OC}}}{n_{\mathrm{id},1}^{\mathrm{rad}}k_{\mathrm{B}}T}\right)}{J_{\mathrm{inj}}(V_{\mathrm{OC}})}\right) = \frac{qV_{\mathrm{OC}}}{n_{\mathrm{id},1}^{\mathrm{rad}}k_{\mathrm{B}}T} - \ln\left(\frac{J_{\mathrm{inj}}(V_{\mathrm{OC}})}{J_{0,1}^{\mathrm{rad}}}\right) \quad \text{(S21)}$$

Rearranging this equation, and noting that $J_{\mathrm{inj}}(V_{\mathrm{OC}}) = J_{\mathrm{ph}}$, gives an expression for V_{OC}

$$V_{\mathrm{OC}} = \frac{n_{\mathrm{id},1}^{\mathrm{rad}}k_{\mathrm{B}}T}{q}\ln\left(\frac{J_{\mathrm{ph}}}{J_{0,1}^{\mathrm{rad}}}\right) - \frac{n_{\mathrm{id},1}^{\mathrm{rad}}k_{\mathrm{B}}T}{q}\ln(Q_e^{\mathrm{EL}}) \quad \text{(S22)}$$

Comparing equation (S22) and equation (S19) we readily find that

$$V_{\mathrm{OC}} = V_{\mathrm{OC}}^{\mathrm{rad}} - \frac{n_{\mathrm{id},1}^{\mathrm{rad}}k_{\mathrm{B}}T}{q}\ln(Q_e^{\mathrm{EL}}) \quad \text{(S23)}$$

Thus, we derive a generalized version of the equation presented in the main text, i.e., an expression for the $\Delta V_{\mathrm{OC}}^{\text{non-rad}}$

$$\Delta V_{\mathrm{OC}}^{\text{non-rad}} = -\frac{n_{\mathrm{id},1}^{\mathrm{rad}}k_{\mathrm{B}}T}{q}\ln(Q_e^{\mathrm{EL}}) \quad \text{(S24)}$$

We reminisce that $n_{\mathrm{id},1}^{\mathrm{rad}}$ is the constant radiative ideality factor of the transitions dominating the emission spectrum, and in our case equal to 1.

Through this treatment, we derive that states that dominate emission at V_{OC} and radiative recombination at $V_{\mathrm{OC}}^{\mathrm{rad}}$ also determine $n_{\mathrm{id},1}^{\mathrm{rad}}$ following the many-diode model. Note, since sub-bandgap states generally have a higher ideality factor than above-gap states, above-gap states dominating emission at V_{OC} will always dominate radiative recombination at $V_{\mathrm{OC}}^{\mathrm{rad}}$ since $V_{\mathrm{OC}}^{\mathrm{rad}} >$

V_{OC} . This result explains why voltage calculations with excellent correspondence to experimentally determined voltages have been achieved in literature for defect-containing active layers without including an $n_{\mathrm{id},1}^{\mathrm{rad}}$ term in equation (S24).

Supplementary References

1 Krückemeier, L., Rau, U., Stolterfoht, M. & Kirchartz, T. How to report record open-circuit voltages in lead-halide perovskite solar cells. *Adv. Energy Mater.* **10**, 1902573 (2020).

2 Pysch, D., Mette, A., & Glunz, S. W. A review and comparison of different methods to determine the series resistance of solar cells. *Sol. Energy Mater. Sol. Cells* **91**, 1698–1706 (2007).

3 Rau, U. Reciprocity relation between photovoltaic quantum efficiency and electroluminescent emission of solar cells. *Phys. Rev. B* **76**, 085303 (2007).
