# Supplementary material for: Effect of sub-bandgap defects on radiative and non-radiative open-circuit voltage losses in perovskite solar cells
Source: Nat Commun. 2024 Feb 10;15:1276. doi: 10.1038/s41467-024-45512-8 (PMC10858920; doi:10.1038/s41467-024-45512-8)
Supplement: Supplementary file 3 — Description of Additional Supplementary Files [file 41467_2024_45512_MOESM3_ESM.pdf]

Description of Additional Supplementary Files

File name: Supplementary Code 1

Description: The Supplementary Code 1 calculates the radiative ideality factor at every photon energy ($n_{\mathrm{id}}^{\mathrm{rad}}(E)$) according to Equation (7) using

- MATLAB script (many_diode_model.m)
- Non-normalized highly sensitive photocurrent spectrum (EQE.txt)
- Normalized and Jacobian transformation-corrected electroluminescence spectrum (EL.txt)
- Current-voltage data of perovskite solar cell in the dark (IV_dark.txt)
- Current-voltage data of perovskite solar cell under 1 sun illumination (IV_light.txt)
- Solar photon flux (AM15G.dat)
