# Supplementary material for: Effect of sub-bandgap defects on radiative and non-radiative open-circuit voltage losses in perovskite solar cells
Source: Nat Commun. 2024 Feb 10;15:1276. doi: 10.1038/s41467-024-45512-8 (PMC10858920; doi:10.1038/s41467-024-45512-8)
Supplement: Supplementary file 5 — Reporting Summary [file 41467_2024_45512_MOESM5_ESM.pdf]

nature research

Corresponding author(s): Rene Janssen

Solar Cells Reporting Summary

Nature Research wishes to improve the reproducibility of the work that we publish. This form is intended for publication with all accepted papers reporting the characterization of photovoltaic devices and provides structure for consistency and transparency in reporting. Some list items might not apply to an individual manuscript, but all fields must be completed for clarity.

For further information on Nature Research policies, including our data availability policy, see Authors & Referees.

Experimental design

Please check: are the following details reported in the manuscript?

1. Dimensions

Item	Reported	Details
Area of the tested solar cells	[x] Yes [ ] No	Methods: Device fabrication
Method used to determine the device area	[x] Yes [ ] No	Methods: Current density – voltage characteristics

2. Current-voltage characterization

Item	Reported	Details
Current density-voltage (J-V) plots in both forward and backward direction	[x] Yes [ ] No	Figure 1b
Voltage scan conditions<br>*For instance: scan direction, speed, dwell times*	[x] Yes [ ] No	Methods: Current density – voltage characteristics
Test environment<br>*For instance: characterization temperature, in air or in glove box*	[x] Yes [ ] No	Methods: Current density – voltage characteristics
Protocol for preconditioning of the device before its characterization	[x] Yes [ ] No	No preconditioning was applied. See Methods: Current density – voltage characteristics
Stability of the J-V characteristic<br>*Verified with time evolution of the maximum power point or with the photocurrent at maximum power point; see ref. 7 for details.*	[ ] Yes [x] No	Cells were stable during EQE, EL and PL measurements.

3. Hysteresis or any other unusual behaviour

Item	Reported	Details
Description of the unusual behaviour observed during the characterization	[x] Yes [ ] No	The solar cells show minimal hysteresis. Stated in the first paragraph of the results section.
Related experimental data	[x] Yes [ ] No	Figure 1b

4. Efficiency

Item	Reported	Details
External quantum efficiency (EQE) or incident photons to current efficiency (IPCE)	[x] Yes [ ] No	Figure 1b
A comparison between the integrated response under the standard reference spectrum and the response measure under the simulator	[x] Yes [ ] No	Stated in the first paragraph of the results section
For tandem solar cells, the bias illumination and bias voltage used for each subcell	[ ] Yes [x] No	No tandem cells were studied.

5. Calibration

Item	Reported	Details
Light source and reference cell or sensor used for the characterization	[x] Yes [ ] No	Methods: Current density – voltage characteristics
Confirmation that the reference cell was calibrated and certified	[x] Yes [ ] No	Methods: Current density – voltage characteristics

Calculation of spectral mismatch between the reference cell and the devices under test

- [ ] Yes
- [x] No

Short-circuit current densities measured match well with integrated EQE response.

6. Mask/aperture

Size of the mask/aperture used during testing

- [x] Yes
- [ ] No

Shadow masks of 0.0676 or 0.1296 cm2 were used. See Methods: Current density – voltage characteristics

Variation of the measured short-circuit current density with the mask/aperture area

- [ ] Yes
- [x] No

One aperture (0.0676 or 0.1296 cm2) for each cell area (0.09 or 0.16 cm2.) was used.

7. Performance certification

Identity of the independent certification laboratory that confirmed the photovoltaic performance

- [ ] Yes
- [x] No

No independent certification of the efficiencies was performed. The efficiencies reported are in line with expectations and were not the focus of this study.

A copy of any certificate(s)
Provide in Supplementary Information

- [ ] Yes
- [x] No

Not applicable

8. Statistics

Number of solar cells tested

- [x] Yes
- [ ] No

25 as stated in the first paragraph of the results section.

Statistical analysis of the device performance

- [x] Yes
- [ ] No

First paragraph of the results section and Supplementary Figure 1.

9. Long-term stability analysis

Type of analysis, bias conditions and environmental conditions
For instance: illumination type, temperature, atmosphere humidity, encapsulation method, preconditioning temperature

- [ ] Yes
- [x] No

Stability was not tested.
